# SDF-1 gene polymorphism and CCL3L1 gene copy number and susceptibility to HIV-1 / AIDS among Indians

**DOI:** 10.1186/1471-2334-12-S1-P50

**Published:** 2012-05-04

**Authors:** Suhani Almal, Anuroopa Gupta, Harish Padh

**Affiliations:** 1 Department of Cellular and Molecular Biology, B. V. Patel Pharmaceutical Education and Research Development (PERD) Centre, Ahmedabad, India

## Background

Stromal derived factor (*SDF-1*) is a natural ligand for CXCR4 and chemokine (C-C) motif ligand 3-like 1 (*CCL3L1*) is for CCR5 HIV coreceptors. The individual role of the SNP in 3′ untranslated region of SDF-1 (*SDF1-3′A*) and low copy number of the *CCL3L1* gene in determining susceptibility to HIV infection is well documented. The aim of the present study was to analyze the synergistic effect of the SNP in *SDF-1* gene and CNV in *CCL3L1* gene influencing the susceptibility to HIV-1/AIDS in Indians.

## Methods

This study involved the assessment of 105 healthy control individuals and 44 HIV-1 patients for the *SDF-1* gene polymorphism by PCR-restriction fragment length polymorphism (RFLP) and *CCL3L1* gene copy number (CN) by real-time PCR.

## Results

In order to assess the synergistic effect of the SDF1-3′A polymorphism and CCL3L1 CN, SDF1-3′A allele and CCL3L1 > 2 copies conferring a protection to HIV-1 were considered as reference combination. The odds ratio (OR) was 0.97 (95% CI - 0.30 to 3.13; *p* = 0.597) for SDF1-3′A, CCL3L1 < 2 copies, 0.73 (95% CI - 0.23 to 2.33; *p* = 0.408) for SDF1, CCL3L1 > 2 copies and 0.93 (95% CI - 0.327 to 2.703; *p* = 0.555) for SDF1-3′A, CCL3L1 < 2 copies combinations as compared to SDF1-3′A, CCL3L1 > 2 copies.

## Conclusion

Our analyses suggest that a combination of SDF1-3′A and lower copy number of *CCL3L1* do not provide any discernible synergistic protection from HIV-1 infection.

